# Echogenic alteration in the raphe nuclei measured by transcranial sonography in patients with Parkinson disease and depression

**DOI:** 10.1097/MD.0000000000013524

**Published:** 2018-12-14

**Authors:** Xue Jiao Liu, Li Zhang, Yong Fang Zhang, Wen Xu, Yang Hu, Ying Liu, Jing Bai

**Affiliations:** a Department of Neurology, Tianjin 4th Centre Hospital, Tianjin; b Department of Neurology, Nanyang Central Hospital, Nanyang; c Department of Neurology, The First Hospital of Jilin University, Changchun; d Wuxi No. 2 People's Hospital, Wuxi, Jiangsu, China.

**Keywords:** depression, Parkinson disease, platelet serotonin concentration, raphe nuclei, transcranial sonography

## Abstract

**Background:**

Recently, several studies using transcranial sonography (TCS) have demonstrated reduced echogenicity of the mesencephalic midline in unipolar depression and patients with comorbid depression and Parkinson disease (PD). However, there is no consensus on the conclusion that raphe nuclei (RN) hypoechogenicity is associated with depression in PD. The methods used in previous studies lack quantitative and objective indicators to some extent; therefore, the present study used the level of platelet 5-hydroxytryptamine (5-HT) as an objective indicator of depression. Additionally, the reason for the reduced echogenicity of the brainstem raphe is still unclear.

**Objectives:**

The purpose of the present study was to assess the correlation between alterations in RN echogenicity and depressive symptoms in patients with PD using transcranial sonography (TCS). This information could provide a meaningful clinical reference for the antidiastole between depressive symptoms in PD and unipolar depression in patients with PD in whom depressive symptoms occur before motor symptoms.

**Methods:**

TCS was performed in patients with PD, patients with PD and depression, patients with depression and no PD, and healthy controls. Using the red nucleus as a reference, the RN was rated from grades 0 to 1 (grade 0: invisible, slightly echogenic, or interrupted RN; grade 1: hyperechogenicity in the RN observed as a continuous line).

**Results:**

The rate of abnormal RN (grade 0) was found to be 16.67% in patients with PD (5/30) and 14.29% in healthy controls (4/28). The presence of abnormal RN was significantly higher (*χ*^2^ = 15.983, *P* < .05) in patients with depression and PD (40%, 12/30) and in patients with depression only (58.33%, 14/24) than in those without depression and healthy controls. No correlation was found between RN changes and depression severity (*P* > .05). There were no statistical differences in the concentration of platelet serotonin among the 4 groups (*P* > .05).

**Conclusions:**

TCS of the mesencephalic midline may be useful for detecting depression, which is an early symptom of PD. However, further neuropathological studies are needed to understand the principles underlying the use of platelet serotonin as a peripheral biomarker, as well as the connection between PD and depression.

## 1 Introduction

Depression is the most common nonmotor symptom in Parkinson disease (PD), affecting 40% to 50% of patients with PD.^[1]^ The risk of PD in patients with depression is 2.2 to 3.1 times higher than that in controls without depression. Some studies suggest that depression is an early symptom of PD.^[2–4]^ However, the depressive symptoms of patients with PD are often ignored because of their motor symptoms. Compared with early motor symptoms, depressive symptoms more strongly influence the lives of patients and cause a serious burden for the family and society. The current view is that depressive symptoms in PD often occur before motor symptoms. As a result, these patients are easily misdiagnosed with unipolar depression. Therefore, the early identification of depression is of great significance for the diagnosis of PD; it is particularly important for the differential diagnosis of Parkinson disease and unipolar depression.

Becker et al^[5]^ were the first to evaluate brainstem changes in patients with PD and depression using transcranial sonography (TCS), and they found that midbrain raphe hypoechogenicity was frequently found in such patients. This was later confirmed by several studies.^[5–11]^ However, the conclusion of 1 study was different from that of the others.^[12]^ Therefore, the purpose of our study was to further assess alterations in RN echogenicity using TCS in patients with PD, depression, and the combination of both.

Though the exact cause of the hypoechogenicity of the RN has not been completely elucidated, changes in raphe echogenicity reflect changes in tissue impedance and point toward an alteration in the brainstem microarchitecture, which could be due to a shift in tissue cell density, change in interstitial matrix composition, or alteration in fiber tract integrity.^[13]^

TCS is a reliable method for the differential diagnosis of parkinsonism because it combines findings from the substantia nigra (SN), basal ganglia, and ventricular system. Moreover, several studies support SN hyperechogenicity as one of the most important risk factors for PD. The advantages of TCS include short investigation time, low cost, and lack of exposure to radiation. However, TCS has its own limitations, which include its dependency on the bone window and operator experience. In the past, studies on the correlation between RN echogenicity and depression in patients with PD only used the Hamilton Depression Rating Scale (HAMD) and cerebral ultrasound, which are both relatively subjective methods. Therefore, to further and more precisely assess the hypothesis that PD-related depression is associated with the hypoechogenicity of the RN, this study added platelet serotonin as an objective, quantifiable indicator of depressive symptoms.

The serotonin low function hypothesis is generally accepted regarding the pathogenesis of depression. This hypothesis states that patients with depression have less serotonin within their central nervous system. Magnetic resonance imaging and postmortem studies have provided evidence that serotonin levels in the central nervous system of depressed patients are reduced.^[14]^ Some studies^[15,16]^ have confirmed that the concentration of 5-hydroxyindoleacetic acid (5-HIAA) in the cerebrospinal fluid of patients with depression is lower than in healthy controls. In clinical studies, however, cerebrospinal fluid is difficult to access; therefore, peripheral indicators of 5-hydroxytryptamine (5-HT) function are urgently needed. Because of concerns that platelet serotonin uptake and release mechanisms are indicative of the activity of 5-HT neurons in the central nervous system (CNS), several researchers have suggested that platelet serotonin levels can be used as a peripheral biomarker for depression or other psychiatric diseases. Bianchi et al^[17]^ advocated that the use of the concentration of platelet serotonin is an effective method for determining the concentration of serotonin in the central nervous system. Unfortunately, however, there is no consensus on whether platelet serotonin levels can be used as a peripheral biomarker of depression. In the present study, we explored the correlation between the reduction of platelet serotonin levels and depressive symptoms.

## 2 Materials and methods

### 2.1 Participants

The participants of the current study were inpatients and outpatients who visited our hospital from November 2015 to December 2017. The presence of PD was determined using the criteria of the United Kingdom Parkinson's Disease Society Brain Bank.^[18]^ The disease stage was established using the Hoehn and Yahr (H-Y) staging system.^[19]^ Patients with unipolar depression were recruited from the psychological health department. According to the criteria of the HAMD, patients with depression were divided into 3 groups using the following cutoffs: an HAMD Rating Score of 8 to 17 for mild depression, 18 to 24 for moderate depression, and ≥25 for severe depression.^[20]^ Sex- and age-matched healthy controls were recruited from our hospital staff and their families and friends. Altogether 112 participants were included in the present study: 24 patients with depression without PD (D+PD−), 30 patients with PD and concomitant depression (D+PD+), 30 patients with PD without depression (D-PD+), and 28 age-matched controls without any psychiatric or neurodegenerative disorders (D-PD−).

The exclusion criteria for controls were the presence of PD and depression according to standard criteria. Patients with essential tremor and those with secondary PD and other Parkinson-plus syndromes were excluded. Subjects with neurological or psychiatric disorders or a family history of such disorders were excluded. Patients with dementia were identified using the Mini-Mental State Examination (MMSE).^[21]^ The exclusion criteria also included the use of controlled substances and the presence of major diseases. Subjects with insufficient bone windows were also excluded from the study.

### 2.2 Data collection methods

#### 2.2.1 TCS

**Figure 1:**
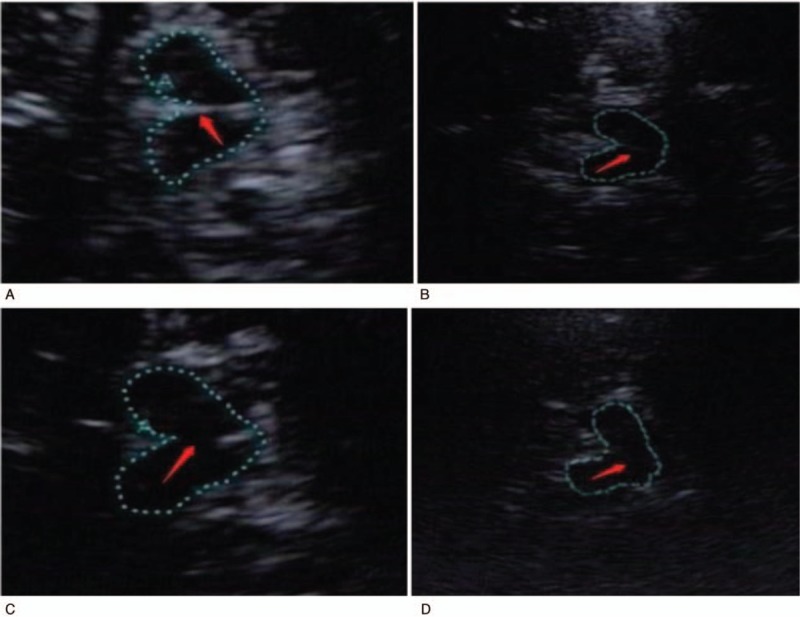
A–D, The echogenicity of the raphe nuclei. A: normal RN; B: slightly echogenic but continuous RN; C: interrupted RN; D: invisible RN.

**Figure 2:**
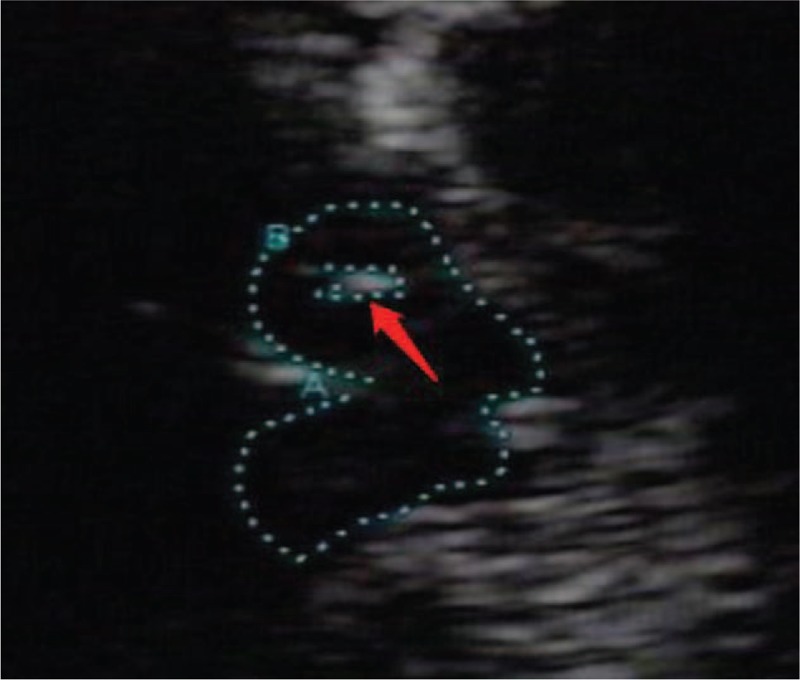
The hyperechogenicity of substantia nigro.

TCS was performed with an ultrasound system (Aplio500; Toshiba, Tokyo, Japan) equipped with a 2.5-MHz transducer. The ultrasound parameters chosen were a penetration depth of 14 to 16 cm and a dynamic range of 45 to 50 dB. The use of dichotomy is recommended internationally. According to this, the echogenicity of the mesencephalic midline raphe was rated on a 2-point scale (grade 0: invisible, slightly echogenic, or interrupted RN; grade 1: hyperechogenicity of the RN observed as a continuous line; Fig. 1A–D).^[22]^ The red nucleus was used as the reference for the rating scale. The examiner determined RN echogenicity by scanning the temporal bone window on the intact skull from both sides. If the RN was depicted as a continuous line from one side, it was rated as normal (hyperechogenicity and noninterrupted midline). Additionally, SN hyperechogenicity and the third ventricle width were analyzed. Areas with SN echogenicity ≥ 0.20 cm^2^ on both sides were classified as hyperechogenic (Fig. 2).^[23]^ It should be further noted that age should be considered a factor when assessing the width of the third ventricle.

All TCS assessments were performed by an expert with 10 years of experience in brain ultrasound who was blinded to the clinical data. The current study was approved by the ethics committee of our hospital (the First Hospital of Jilin University), and informed consent was obtained from each participant.

#### 2.2.2 Platelet serotonin levels

Five milliliter of venous blood from each subject were drawn on an empty stomach at 6:00 am. After collecting and centrifuging blood in anticoagulant tubes, plasma containing platelets was obtained. Plasma samples were then placed in a −20^o^C refrigerator before enzyme-linked immunosorbent assay (ELISA) was performed to obtain the concentration of platelet serotonin in all subjects. The determination of the concentration of platelet serotonin was the responsibility of professional and technical personnel of the Weixuan Biotech Company.

### 2.3 Statistical analysis

Data were analyzed using SPSS 16.0 software (IBM Corp, Armonk, NY). Descriptive statistics are presented as means and standard deviations. *χ*^2^ tests were performed to assess correlation between changes in platelet serotonin and depression severity in the 4 groups. The ratios of RN abnormalities in the 4 groups were also analyzed using the *χ*^2^ test. Spearman rank correlation analysis was used to assess correlation with gender, age, and abnormal RN echogenicity. We obtained differences in platelet serotonin concentration among the 4 groups using variance analysis. Statistical significance was set at *P* < .05.

## 3 Results

### 3.1 Clinical characteristics

Initially, 155 patients were recruited for the current study. Twenty-three (14.8%) patients were excluded due to insufficient temporal bone windows. Of the 132 remaining, 7 were also excluded because of MMSE scores below 24. Four participants were excluded due to errors in the determination of their platelet serotonin concentration. In the end, the current study included 60 patients with PD (30 D+PD+, 30 D-PD+), 24 patients with depression only, and controls. The results of the analyses of clinical characteristics are provided in Table 1. There were no significant differences in sex or age in the 4 groups, and there were no significant differences in the duration of PD or depression, or in the H-Y stage in patients with PD (*P* > .05).

**Table 1:**
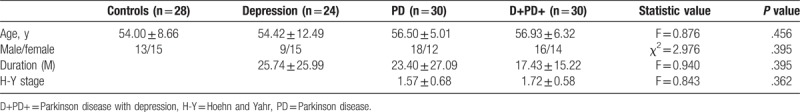
The results of the analyses of clinical characteristics.

### 3.2 Platelet serotonin levels

In the current study, no statistical significance was found between age, gender, duration, H-Y stage or treatment drugs, and platelet 5-HT concentration (*P* > .05). Further, we did not find any associations between depressive symptoms in PD and age, gender, PD duration, H-Y stage, or 5-HT concentration (Tables 2 and 3).

**Table 2:**
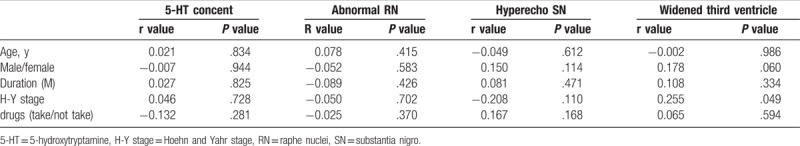
Factors affecting the 5-HT concentration and RN echogenicity.

**Table 3:**
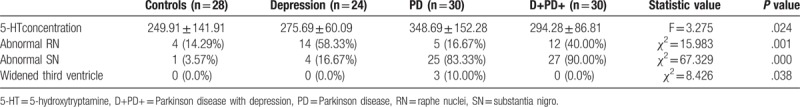
Factors affecting depression.

### 3.3 RN abnormalities measured by TCS

We found that the rate of abnormal RN echogenicity (Grade 0) in D+PD+ patients and D+PD− patients was much higher than in the PD+D− and control groups (40%, 58.33%; 16.67%, 14.29%). This difference was statistically significant (*χ*^2^ = 15.983, *P* < .05; Table 3). However, no correlation was found between RN changes and depression severity (*P* > .05; Table 4). Further, there was no association between SN hyperechogenicity and RN hypoechogenicity (r = 0.110, *P* = .252).

**Table 4:**
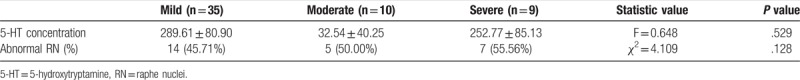
Correlation between RN echogenicity and depression severity.

### 3.4 SN hyperechogenicity and the broadening of the third ventricle

The rates of SN hyperechogenicity were statistically different between groups. When compared with subjects with normal SN echogenicity, most D+PD+ and D-PD+ patients exhibited SN hyperechogenicity (*P* < .05). Some studies have suggested a relationship between the width of the third ventricle and depression.^[24,25]^ Unfortunately, we were not able to confirm these findings. We found hyperechogenicity of the SN in 4 of the patients in the D+PD− group. According to the hypothesis that depression might be an early clinical manifestation of PD,^[26]^ follow-up of these patients might help in the early diagnosis of PD.

## 4 Discussion

Many studies have used TCS to explore the echogenicity of the RN.^[5–11]^ However, there is no consensus on whether this is a useful and accurate method of investigation. Some studies have found that patients with concomitant PD and unipolar depression have decreased echogenicity of the RN compared with subjects with only PD or healthy controls.^[5,8–10]^ However, Bouwmans et al^[12]^ reported that they did not find any relationship between echogenicity of RN and depression in patients with or without Parkinson disease. The results of the current study are consistent with previous findings that subjects with depression and PD as well as patients with unipolar depression have decreased echogenicity of the RN compared with subjects with PD only or healthy controls.

Moreover, no correlation was found between RN differences and depression severity in the current study, which contradicts the results of Zhang et al.^[7]^ However, compared with the mild depression group, the rate of abnormal RN in the severe depression group was significantly higher. The difference between both groups was statistically significant (*χ*^2^ = 4.095, *P* = .043).

Additionally, postmortem studies and MRI have demonstrated that abnormal RN echogenicity reflects destruction of serotonergic neurons of the brainstem raphe. This phenomenon results in a decrease in 5-HT levels in the CNS. However, there is no consistent conclusion regarding the association between platelet 5-HT and 5-HT in the CNS. It is also not clear whether platelet 5-HT levels can be used as a peripheral biochemical marker of depression. In the current study, no statistically significant correlation was found between platelet serotonin and depression, or between platelet serotonin and abnormal RN echogenicity. This might be because abnormality of the raphe nucleus is not only due to reduction in number of 5-HT neurons. We suggest that caution be exercised when using platelet serotonin as an indicator of the activity of 5-HT-neurons. Interestingly, we were unable to find a correlation between abnormal RN and PD severity. This contradicts results reported in previous studies, which suggested that patients with abnormal echogenicity display more advanced H-Y stages than those with normal RN echogenicity.^[9]^

Some of the aforementioned conclusions are not consistent with the results of some previous studies. This may be due to some limitations in the present study. First, the sample size of the current study was relatively small. Further, there was no investigation or follow-up study before or after administration of serotonin reuptake inhibitors. Additionally, we only used 1 scale to evaluate depression.

## 5 Conclusion

As marked reductions in RN echogenicity in patients with PD and depression as well as patients with depression and no PD were found in our study, TCS might be helpful in the diagnosis of depression, which is an early symptom of PD. However, we were unable to find a relationship between RN hypoechogenicity and depression severity. If a patient with depressive symptoms has both RN hypoechogenicity and SN hyperechogenicity, they might tend to be diagnosed with PD rather than unipolar depression. Moreover, no statistically significant correlation was found between the concentration of platelet serotonin and depression. There was also no correlation between serotonin levels and RN echogenicity. Further studies with larger sample sizes and studies investigating the underlying pathological mechanisms of the relationship between PD and depression are needed.

## Acknowledgments

The authors want to thank their many colleagues who helped to procure the clinical specimens and Transcranial sonography technicians who helped complete the experiments.

## Author contributions

**Conceptualization:** Xuejiao Liu.

**Data curation:** Yang Hu.

**Formal analysis:** Jing Bai, Li Zhang.

**Resources:** Yongfang Zhang, Wen Xu.

**Software:** Ying Liu.

**Supervision:** Jing Bai.

**Writing – original draft:** Xuejiao Liu.
